# Supplementary material for: Effect of Sodium Hyaluronate on Antioxidant and Anti-Ageing Activities in Caenorhabditis elegans
Source: Foods. 2023 Mar 26;12(7):1400. doi: 10.3390/foods12071400 (PMC10093893; doi:10.3390/foods12071400)
Supplement: Supplementary file 1 [file foods-12-01400-s001.zip › foods-2238948-supplementary.pdf]

Supplementary Material

Table S1 Effect of SH on prolonging the lifespan of *C. elegans*

Group	N	Mean lifespan（d）	% Of control	Maximum lifespans（d）
Control	90±1	15.33±0.58^{a}	100.0^{a}	19.67±1.15^{a}
200μg/mL	89±2	16.83±0.29^{b}	109.86±3.54^{b}	23.67±0.58^{b}
400μg/mL	91±2	18^{c}	117.50±4.33^{c}	25±1.73^{c}
800μg/mL	92±1	18±1^{c}	117.50±7.95^{c}	28.33±1.15^{c}

Data were showed in mean ± SD, n = 3. Values with different letters in the same column indicate statistical significance (p<0.05)

Table S2 Effects of SH on the fecundity of *C. elegans*(N=5)

Concentration (μg/mL)	Experimental group（N=5）					Mean ± SD
	1	2	3	4	5	
0	310	315	309	300	309	308±6^{a}
200	337	331	316	308	323	323±13^{a}
400	312	317	326	314	321	318±5^{a}
800	303	318	334	308	319	316±11^{a}

*Data were showed in mean ± SD, n = 5. Note: Mean number of eggs laid is rounded and values with different letters in each group indicate: a significant difference between the two groups (p<0.05)

Table S3 Protective effect of SH on heat stress injury of *C. elegans*

Group	N	Mean lifespan（h）	% Of control	Maximum lifespans（h）
Control	91±1	8.0^{a}	100.0^{a}	13.33±2.31^{a}
200μg/mL	91±2	9.67±0.58^{ab}	120.83±7.22^{ab}	16.67±3.01^{b}
400μg/mL	87±3	10^{b}	125.0^{b}	15.33±1.15^{c}
800μg/mL	89±1	10^{b}	125.0^{b}	16.67±1.54^{c}

Data were showed in mean ± SD, n = 3. Values with different letters in the same column indicate

statistical significance (p<0.05)

Table S4 Protective effect of SH on H_2O_2 stress of *C. elegans*

Group	N	Mean lifespan（h）	% Of control	Maximum lifespans（h）
Control	89±1	6.0[a]	100.0[a]	12.67±1.15[a]
200μg/mL	91±4	7.33±0.58[ab]	122.21±9.6[ab]	14.33±0.58[ab]
400μg/mL	87±2	7.67±0.58[ab]	127.78±9.6[ab]	14.67±1.15[ab]
800μg/mL	90±1	11.67±1.15[c]	194.45±19.27[b]	17.33±1.55[b]

Data were showed in mean ± SD, n = 3. Values with different letters in the same column indicate statistical significance (p<0.05)

Table S5 List of used for rt-PCR assays

Gene's name	Forward (5′-3′)	Reverse (5′-3′)
β-actin	GCCGTGTTCCCATCCATTGT	CCTCTCTTGGATTGGGCCTC
daf-2	TGAAAGCGAAGCAGGAGAAGG	CGTCCGAACTTCCGCATCACTC
daf-16	GCGGAGCCAAGAAGAGGATA	GTTCGGGGACGGAAAGATGA
sod-3	ATCACTATTGCGGTTCAAGGCTCTG	TTGCACAGGTGGCGATCTTCAAG
gst-4	TTGATGCTCGTGCTCTTGCT	CGTTGGCTTCAGCTTTGACC
shn-1	GGTCTCCGTTGGCGTGATGATC	CTGGTGGATGCTCGGTGAGTATTG

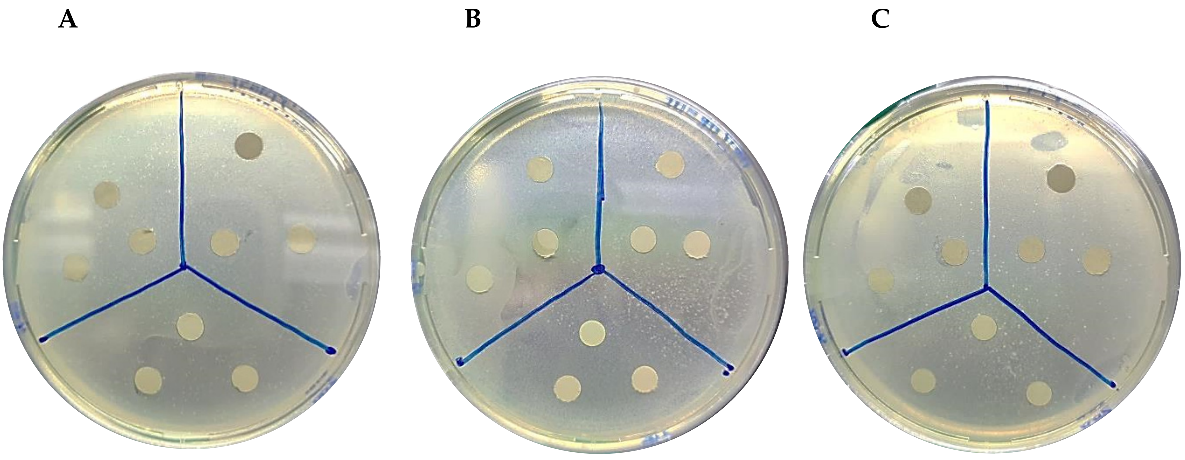

a. 200 μg/mL　　b. 400 μg/mL　　c. 800 μg/mL

Figure S1 Drug sensitivity test of SH against Escherichia coli OP50. The three plates shown are all NGM (d=90 mm); each plate was divided equally into three parts, 400 μl OP50 was taken and spread evenly on the plate, and the bacterial solution was allowed to dry before being pasted onto circular paper sheets (d=5 mm) containing SH solution. a. 200 μg/ml of SH solubility; b. 400 μg/ml of SH concentration; c. 800 μg/ml of SH concentration. In each group, all the paper sheets were surrounded by the appearance of inhibition circles.
